# The extent of interruptions to primary care medical officers’ consultations in the Western Cape

**DOI:** 10.4102/safp.v66i1.5957

**Published:** 2024-07-17

**Authors:** Tsepo S. Motsohi, Bob Mash, Michael Pather, Louis Jenkins, Paul Kapp, Johannes F. Schoevers, Mumtaz Abbas, Leigh Wagner, Salome Froneman, Stefanie Perold, Gavin D. Hendricks

**Affiliations:** 1 Division of Family Medicine and Primary Care, Faculty of Medicine and Health Sciences, Stellenbosch University, Cape Town, South Africa; 2 Department of Health and Wellness, Bishop Lavis CDC, Cape Town, South Africa

**Keywords:** consultation interruptions, primary care administrative burden of medical officers, cognitive load, Western Cape rural and urban health, clinic doctors

## Abstract

**Background:**

Administrative tasks are an increasing burden for primary care doctors globally and linked to burnout. Many tasks occur during consultations. They cause interruptions with possible effects on patients’ and doctors’ experiences and care. The burden and typology of interruptions of doctors in primary care consultations have not been studied in South Africa. Given the link between administrative loads and burnout, describing the extent of these interruptions would help. This study’s aim was to assess the extent of interruptions on primary care doctors in the Western Cape.

**Methods:**

This was a descriptive cross-sectional survey. Doctors from rural and urban primary care clinics in the Western Cape answered an online self-administered survey on the types of interruptions experienced during consultations. Interruptions were categorised and their prevalence calculated. Clinical and non-clinical interruption categories were compared.

**Results:**

There were 201 consultations from 30 doctors. Most interruptions were from retrieving and recording the current patient’s information (93.0%), paperwork for other patients (50.7%), and telephone calls about the current patient (41.8%). Other prevalent interruptions were for emergencies (39.8%) and acquiring consumables (37.3%). The median (interquartile range [IQR]) of four (2–4) interruption types per consultation was higher than global settings.

**Conclusion:**

Doctors experienced many interruptions during consultations. Their wide range included interruptions unrelated to the current patient.

**Contribution:**

This study adds insights from the global south on clinicians’ administrative burden. It elaborates on the types of activities that interrupt consultations in an upper-middle income primary care setting. Exploration of interventions to decrease this burden is suggested.

## Introduction

Administrative work has been a part of doctors’ responsibilities for many years. However, these responsibilities have increased globally, with the establishment of various essential clinical governance and reporting standards, as well as legal corporate governance requirements embedded in both private and public health systems.^1,2,3^ In ambulatory primary care, clinicians have been found to spend up to 49.2% of their work days on administrative tasks and completing electronic data records.^4^ These tasks include completing insurance-related or worker’s compensation forms, obtaining prior authorisation for medications, tests, reviewing other patient folders, and preparing reports.^4,5,6^ The increasing burden of administrative tasks has been demonstrated to be a significant contributor to clinician burnout, particularly in primary care.^3,6^ In a recent study, increasing administrative tasks and responsibilities were one of the contributing reasons for general practitioners’ leaving the National Health Service in England earlier than expected.^1^

Many of these administrative activities are conducted before or after consultation times and are facilitated by administrative support staff and software.^2,3,5^ However, administrative activities still feature significantly during consultations with patients.^3,5^ During consultations, they invariably disrupt the interaction, with possible effects on the patient’s and doctor’s experience and care provided. Of course, administrative tasks are only one of numerous reasons why clinical care is interrupted.^7,8,9,10^ These reasons range from telephone calls, smartphone interruptions, entry of third parties, and others.^7,8,9,10^

The cognitive impact of interruptions on performance is a substantial field of study affecting many professions and approached by researchers from diverse disciplines.^11,12,13,14^ The general consensus is that the disruption caused by an interruption is proportional to the complexity of the current (primary) task, the duration of the interrupting task as well as the similarity of the tasks.^11,13,15^ Nonetheless, some tasks are unavoidable and even necessary to the interaction with the patient (e.g., checking laboratory results on the computer, or making a telephone call for clinical advice from another colleague). Rivera and Karsh outline a sociotechnical framework for categorising interruptions based on the positive and negative outcomes for both the interrupter and the interrupted doctor.^10^ The framework incorporates both internal and external causes of interruptions and offers a structure for studies on interruptions to clinical encounters (see Table 1).^10^

**TABLE 1:** Conceptual framework for interruptions in healthcare.

Outcomes	Interrupter	Interruptee	Example
Positive – Positive	Gains wanted information or provides necessary information	Gains necessary information and resumes primary task or appropriately changes task.	The doctor is typing up a prescription for a patient when the computer software alerts him that the patient is allergic to that medication.
Positive – Positive and Negative	Gains wanted information or provides information	Gains necessary information but also forgets to resume primary task.	The nurse is looking for medication for their patient when their pager warns that their other patient is coding. The nurse responds, but subsequently forgets to return to get the medication for their first patient.
Positive – Negative	Gains wanted information or provides information	Distracted, does not resume primary task or resumption is delayed.	The pharmacist is entering orders into the computer system when a nurse asks them how they should administer a new medication to their patient. The pharmacist gets distracted and forgets where they are in the order entry process.
Negative – Negative	Gains the wrong information or does not gain wanted information.	Distracted, does not resume primary task or resumption is delayed.	The nurse interrupts a registrar to ask a question about a medication item. The registrar provides the wrong information and forgets what they were doing originally.
Negative – Neutral	Gains the wrong information or does not gain wanted information.	Distracted, but appropriately resumes primary task.	The nurse interrupts a registrar to ask a question about a medication item. The registrar provides the wrong information, and then resumes their original task.
Neutral – Negative	Does not provide or receive information.	Distracted, does not resume primary task or resumption is delayed.	The nurse is charting and a known false alarm interrupts them and they forget to resume charting.
Neutral – Neutral	Does not provide or receive information.	Distracted, but appropriately resumes primary task.	The nurse is charting and a known false alarm interrupts him, but he resumes charting.

*Source*: Rivera AJ, Karsh BT. Interruptions and distractions in healthcare: Review and reappraisal. Qual Saf Health Care. 2010;19(4):304–312

In South Africa, as the health and patient information systems in primary care have grown, an unintended outcome has been an overload of data requiring collection.^16^ Although there is no research literature on administrative burden of doctors in South Africa, anecdotal evidence suggests that these monitoring and reporting requirements have added an administrative burden on primary care doctors, particularly during consultations. Furthermore, the extent and types of interruptions to the primary care consultation have not been assessed in this setting. Given the evidence that administrative loads are significant contributors to doctors’ attrition and burnout, it would be helpful to assess and describe the extent and nature of interruptions in a primary care South African setting.^2,3,6^ The aim of the study was to assess the extent of interruptions on medical officers (MOs) in primary care settings of the Western Cape, South Africa. Specific objectives included to categorise and measure the common types of clinical and non-clinical interruptions in doctors’ consultations.

## Research methods and design

### Study design

This was a descriptive cross-sectional survey.

### Study setting

The Western Cape is one of nine provinces in South Africa. Based on 2023 data from Statistics South Africa, the province is estimated to have a population of 7.4 million, and approximately 80% are dependent on public health services.^17^ District health services in the province are governed by two directorates, namely the Rural Health Services (RHS) and the Metro Health Services (MHS), both of which are further divided into five districts.^18^ The Stellenbosch University Family Physicians’ Research Network (SUFPREN) includes 31 family physicians in all of these districts.^19^ Table 2 outlines the hospitals and facilities where these family physicians are located. Family physicians in SUFPREN are employed by the Department of Health and Wellness in district hospitals and larger primary care facilities. Applied research is part of their job description and members of the network identify and prioritise research questions. The research question in this study was prioritised by members of SUFPREN as a key issue.

**TABLE 2:** Locations of Stellenbosch University Family Physicians’ Research Network family physicians.

Health service	District/substructure	Number of district hospitals	Number of primary care facilities	Number of family physicians
Rural	Garden Route	3	0	8
	Overberg	1	0	2
	Cape Winelands	3	0	6
	West Coast	1	0	1
Metro	Northern-Tygerberg	0	3	3
	Khayelitsha-Eastern	2	2	9

The district health system in South Africa is a unique working environment where the first ambulatory clinician–patient encounter involves either a doctor, a clinical associate, or a professional nurse (clinical nurse practitioner [CNP]).^20^ The overall design of the district health system involves well-distributed primary care facilities in a catchment area that is supported by a district hospital.^20^ In the Western Cape, the primary care provider in the clinic is a CNP who has received a year’s post-graduate training as a generalist.^20^ The CNPs are supported by a MO who is either based at the primary care facility or the supporting district hospital.^20^

### Study population

The study population included full-time MOs who had been working in the primary care facilities of the RHS and MHS for a minimum of 1 year. This also included MOs who were based at the supporting district hospital but conducted weekly outreach visits to primary care clinics. The MOs were in geographical areas that were covered by the SUFPREN network. Locum MOs, community service doctors and interns were excluded as they were temporary members of staff (< 1 year).

### Sample size calculation

The sampling unit was the primary care consultation, and to calculate the sample size, the estimated prevalence of non-clinical interruptions was estimated as 10%, with a margin of error of 5% and normally distributed 95% confidence interval.^4^ Non-clinical interruption prevalences in the literature were lower than clinical interruptions.^4,6^ The size of the study population was assumed to be 20 000 as above this the sample size does not change substantially. Given these assumptions, the required sample size was 139 consultations.

### Study sampling

Medical officers from each of the facilities included in the SUFPREN network were approached for recruitment. Nine of the SUFPREN family physicians approached and recruited MOs from all the possible MOs working in their areas of responsibility.^4^ Medical officers were requested to randomly sample two or three consultations a day over a period of 2 weeks. This was meant to accommodate both facility-based MOs and outreach MOs who visited a facility once or twice a week.

### Data collection

The literature review and identification of key issues by the SUFPREN four researchers were used to design the questionnaire.^4,5,21,22^ For the purposes of this study, three categories of interruption types were defined as follows:

- Non-clinical interruptions:
  ■ Personal breaks and interactions not related to work (e.g., toilet, phone calls, texting, e-mails).
  ■ Other non-personal but work-related interactions not related to any specific patient (e.g., announcement about a meeting from a manager or colleague).
  ■ Completion of administrative paperwork (e.g., timesheets, statistics sheets, registers).
- Clinical interruptions related to consultation with index (current) patient:
  ■ Any telephonic, electronic (e-mail, application or others), or face-to-face communication related to the index patient. This included communication with another staff member, student, or referral centre about the patient’s current care. It also included referring patients either telephonically, electronically, via cell phone applications or the writing of referral letters.
  ■ Any patient record documentation (paper or electronic) related to the current patient, including notes, filling prescriptions, ordering tests (X-rays, blood tests, etc.), forensic medicolegal forms, among others.
  ■ Filling in patient-related administrative forms including medico-legal forms, occupational injury forms, insurance forms, registration of death forms, disability grant forms, among others.
- Clinical interruptions not related to consultation with index patient:
  ■ Any telephonic, electronic (e-mail, application, or others), or face-to-face communication related to another patient. This also included any emergencies.
  ■ Entry into the consulting room by a colleague to collect consumables and/or equipment not in their own consulting room.
  ■ Administrative telephonic, online, or paperwork for other patients.

The draft questionnaire was validated by presentation to SUFPREN family physicians during a workshop. Further inputs were invited by e-mail after the workshop. The questionnaire was designed as an online electronic questionnaire using REDCap and had built-in checks for completion. Piloting of the electronic questionnaire was conducted with three MOs, and a family physician at a community health centre in the MHS, and adjustments made on the usability of the online electronic questionnaire, and the clarity and comprehensibility of individual questions.^23^ The pilot consultation data were not included in the study.

The participating MOs completed the self-administered questionnaire on the number and types of interruptions immediately after each consultation. Each MO was requested to randomly select and complete two or three questionnaires a day for a period of 1 week. No additional instructions were provided on which consultations to select.

Participants were requested to indicate the length of time taken by each of the listed interruptions that occurred during the consultation. Demographic information including sex, age, and the MO’s rank were also collected to describe the participants. The MOs’ ranks are defined as follows based on the number of years of employment: grade 1 (0 to 5 years), grade 2 (5 to 15 years), grade 3 (15+ years). The district where each consultation was conducted was also recorded.

### Data analysis

IBM^®^ Statistical Package of Social Sciences (SPSS) Statistics version 29.0.0.0 was used for statistical analysis. In addition to the built-in completion checks in REDCap, questionnaires were checked for completion by the primary investigator. The data were imported to Excel, cleaned further, and then imported to SPSS. Analysis was conducted by the principal investigator and supported by a biostatistician.

For each individual consultation, the total number of interruptions in each category (as defined above) was summated. The proportions of interruptions in each category for that consultation were then calculated. Finally, the overall median proportion of interruptions per category was calculated. In addition, the total duration of all the interruptions in each consultation was calculated and subsequently analysed as a median total interruption time.

Descriptive statistics were used for numerical data to report either means and standard deviations, or medians and interquartile ranges, depending on the distribution. Frequencies and percentages were used for categorical data. The proportion of individual interruption types was calculated using the number of consultations as the denominator. The median duration of each interruption type in all the consultations was also analysed.

### Ethical considerations

The study was approved by the Stellenbosch University Health Research Ethics Committee (HREC approval number N22/06/072) and the Western Cape Provincial Health Research Database Committee (Approval number WC_202210_024). Confidentiality was ensured by storing all data on a password-protected computer. Anonymity was maintained by assigning anonymous numerical identifiers to each participant.

## Results

The sample included 201 consultations from 30 MOs (see Table 3). Most of the MOs (70%) were based at the primary care facility and 30% were performing outreach. These included any family medicine registrars who also perform outreach to primary care clinics as part of the family medicine speciality training. The majority were grade 1 MOs (56.7%) and were female (66.7%). The median number of consultations per MO was 3.5 (interquartile range [IQR]: 1 to 10). The MOs were from the Metro East subdistricts (12 MOs) and the Garden Route subdistricts (11 MOs), while five MOs were from the Overberg subdistricts and two were from the West Coast subdistricts.

**TABLE 3:** Demographics of medical officers (*n* = 30).

Variables	Category	Frequency	%
Gender	Male	10	33.3
	Female	20	66.7
Clinician role	Permanent MO	21	70.0
	Outreach MO	9	30.0
Rank (incl. registrar)	Grade 1	21	56.7
	Grade 2	6	20.0
	Grade 3	3	10.0

incl., including; MO, medical officer.

Overall, 55.2% of the consultations were from the MHS and 44.8% were from the RHS. All districts covered by SUFPREN network were represented by the data except for the Cape Winelands District, which did not provide permission for the study (see Table 4).

**TABLE 4:** Geographical distribution of consultations (*N* = 201).

Variables	Category	Frequency	%
Region	Garden route	47	23.4
	Overberg	22	10.9
	West Coast	21	10.4
	Metro East	111	55.2

**FIGURE 1:**
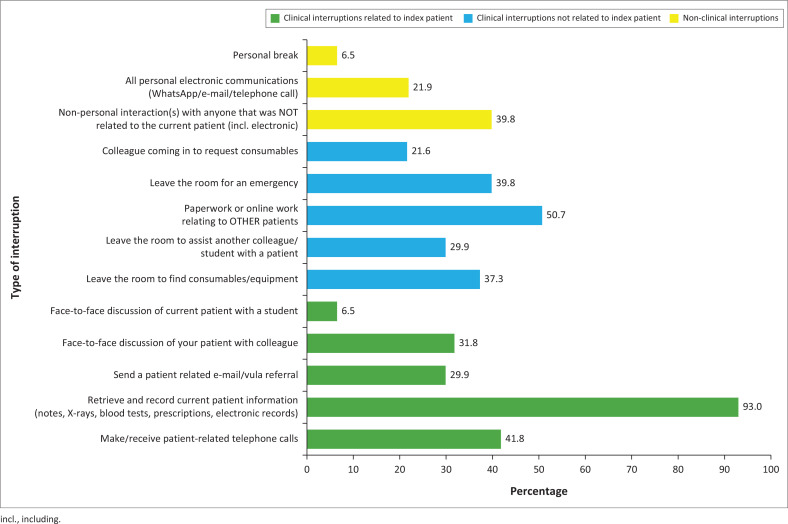
Prevalence of interruptions by consultations (*N* = 201).

Figure 1 presents the prevalence of different types of interruptions in the 201 consultations. All types of interruptions occurred in more than 1 in 5 (20%) consultations, apart from personal breaks (e.g., using the toilet) and discussions with a student. The two commonest interruptions were retrieving and recording the index patients’ information (93%) and paper or online work related to other patients (50.7%). After this, the next three commonest interruptions were making or receiving phone calls about the index patient (41.8%), leaving the room to deal with an emergency (39.8%), and non-personal interactions not related to patients (39.8%).

The median proportion of interruptions related to the current patient was 60% (IQR: 60 to 80) and the median proportion of interruptions related to another patient was 25% (IQR: 0 to 33).

Additionally, the median proportion of non-clinical interruptions was 11% (IQR: 0 to 25).

The median number of total interruptions in a consultation was four (IQR: 2 to 4), while the median number of clinical interruptions related to the current patient was two (IQR: 1 to 3). The median number of clinical interruptions not related to the index patient was one (IQR: 0 to 2) and the median number of non-clinical interruptions was one (IQR: 0 to 1).

**FIGURE 2:**
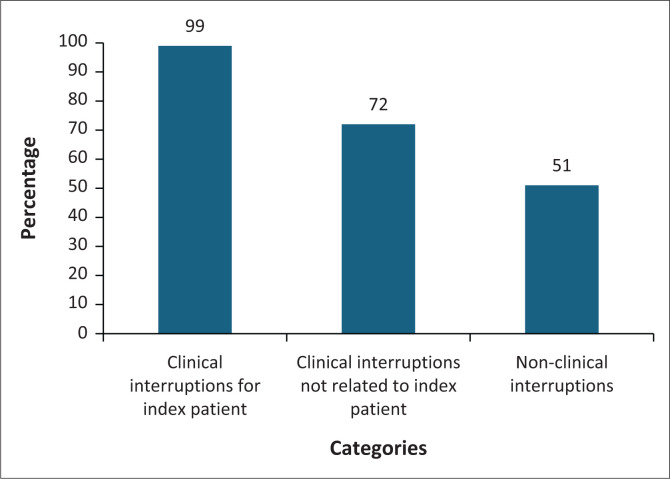
Prevalence of interruptions by categories (*N* = 198).

Figure 2 shows the proportion of consultations that were interrupted according to the categories. Overall, 99% of all consultations were interrupted by issues related to the index patient, 72% of consultations by issues related to other patients, and 51% of consultations with non-clinical issues not related to any patient.

The median duration of all interruptions was 14 min (IQR: 10 to 22) per consultation and the median duration of all the clinical interruptions related to the index patient was 10 min (IQR: 6 to 15). The median duration of all clinical interruptions that were not related to the index patient was 2 min (IQR: 0 to 5), while the median duration for all non-clinical interactions was 1 min (IQR: 0 to 3). For the two commonest types of interruptions, the median length of time to record and retrieve information related to the current patient was 5 min (IQR: 2 to 7), while the median time for interruptions to conduct paperwork or online work for an unrelated patient was 1 min (IQR: 0 to 3).

## Discussion

### Summary of key findings

Most interactions with patients were interrupted four times by a combination of issues related to the patient (99% of consultations), other patients (72% of consultations), and other non-clinical issues (51% of consultations). This has a huge potential for cognitive disruption and risk of errors. The largest amount of time (median 10 min) was spent on activities related to the patient, but not involving actual interaction with them. Inefficiencies in obtaining consumables, communication systems, retrieving and recording patient information may be contributing to avoidable delays resulting in disruption of the doctor–patient relationship and clinical reasoning. The three most prevalent interruptions found in our study were retrieving or recording the current patient’s information (93%), doing paperwork/online work related to other patients (50.7%), and making or receiving telephone calls related to the current patient (41.8%). Although the time spent in interruptions related to other patients (median: 2 min) and non-clinical issues (median: 1 min) was much less, the frequency of such interruptions was likely to be disruptive.

### Discussion of key findings

Primary care consultations are typically very brief, with median lengths ranging from 11 min to 14 min.^7,24^ In the very few studies of interruptions of consultations, the median number of interruptions is one.^9,25,26^ Therefore, a median of four interruptions per consultation as found in our study likely affects the quality of the consultation more, both for the clinician and for the patient. Furthermore, the wider range of median interruption types in our study likely adds to the complexity of the consultation. While many of the clinical interruptions related to the patient are likely to benefit the consultation, the interruptions related to other patients and non-clinical interruptions are likely to have a neutral to negative effect (Table 1).^10^ Interrupting the consultation to assist a colleague or a student with a clinical matter may be a similar category of task to the one in the consulting room (i.e., clinical), but its length is still arguably disruptive.^11^ Furthermore, leaving the consulting room for equipment or interrupting to conduct paperwork for a different patient also takes enough time to cause significant cognitive disruption, thereby increasing the probability of a negative outcome as described by Rivera and Karsh’ framework.^10,11^

The connection between interruptions in the clinical setting and clinical errors has been well-established, particularly within hospital settings like wards and emergency rooms.^10,15,26,27^ In a recent French national study, the incidence of reported patient safety incidents (PSI) in primary care general practice settings was 26 per 1000 patient encounters per week.^28^ More notable was the study’s finding that the incidents were most frequently related to the organisational processes of healthcare rather than clinicians’ knowledge and skills.^28^ These were further categorised to reveal processes occurring within the consultation room itself, including communication, medication, and nonmedication treatment errors.^28^

It is notable that most of the participants (57%) in the study had five or less years of experience. This may be specifically relevant when considering the interruptions because of paperwork related to other patients (50.7% prevalence). It is possible that many of these activities are related to a lack of mentoring on task prioritisation, or organisational support of more junior MOs for protected clinical administrative time. Time management and task prioritisation skills among clinicians have been found to be associated with more years of experience in the workplace, and more notably, they can be taught.^29,30,31^

The interruptions because of paperwork related to other patients could also be related to the lack of electronic equipment like computers and network access in all clinic consultation rooms, resulting in the MOs’ using their computers to complete tasks like searching for online results for their CNP colleagues. More specifically, the interruptions highlight the continuing gaps caused by the lack of an electronic patient health record. A patient electronic health record operating from a single platform with access to a patient’s health information would arguably minimise the disruptive nature of accessing patient information from multiple sources during a consultation. The development of a patient electronic health record has been prioritised by the national department of health as one of the critical features of its electronic Health (eHealth) strategy as it continues the country’s journey to National Health Insurance.^32,33,34^

The latest public service commission report of the state of South Africa’s primary care clinics found that only 18% of the inspected clinics had computers.^35^ This lack of information and communication technology infrastructure is one of the main technical barriers to the implementation of electronic health records in South Africa.^32^

The interruptions because of leaving the room or a colleague’s entering the room for consumables suggest more basic challenges with daily operational management of facilities and possibly, adequate bulk supply management. These issues are a continuing challenge across the primary care system, with many facilities demonstrating improvement through quality improvement interventions that were initiated following the national health department’s ongoing Ideal Clinic Realisation and Maintenance programme.^36^ Although a Portuguese study also found a higher prevalence of interruptions because of leaving to find consumables (21.6%), our study reveals a wider range of interruption types than in the Portuguese and other higher income settings where interruptions of primary care consultations have been studied.^7,8,25^

### Strengths and limitations

This is the first study to describe a detailed typology of interruptions of primary care consultations in an upper-middle income country context. The study has a wide urban and rural coverage and questionnaire completion rate. The ease of the data collection also means that the study can be replicated in a similar setting with minimal resources. It is also the first study in this context to compare the different broad categories of interruptions and provide a description of their comparative burdens on doctors. Recall bias was minimised by requesting participants to upload information about a consultation immediately after it was completed. The short length of the questionnaire combined with a broad range of categories of interruptions helped to minimise any information or measurement bias.

The study is limited by its coverage of only areas within the SUFPREN network and excluded areas not covered by the network in the province. These included the western parts of the Metro District, and the northern parts of the West Coast district. The Cape Winelands district, which is covered by SUFPREN, declined permission for the study to be conducted there, because of the already significant number of research studies being conducted in the area. These areas do not differ substantially in how health services are organised, and the sample includes similar rural and metro facilities. Furthermore, the inherent self-selection bias in the consenting MOs is an unavoidable limitation, as well as each participant’s own potential selection bias when randomly selecting consultations. This could have led to participants’ selecting consultations that were interrupted the most, thereby inflating the prevalence of interruptions, the durations of interruptions, as well as the median number of interruptions per consultation. More training on how to randomise their selection of consultations could have mitigated this bias.

### Recommendations

In their extensive study of the increasing administrative burden on clinicians, Rao et al. provide a helpful analysis of the types and proportions of administrative tasks that can be delegated to non-clinician staff.^2^ A similar approach and analysis could be applicable to the tasks related to some of the interruptions of consultations in our study. Many of the interruptions are avoidable. The most obvious is the leaving of the consulting room to collect consumables or equipment related to the current patient consultation. Routine, comprehensive preparation of consulting rooms is a nursing assistant function that could prevent such interruptions, including those because of colleagues’ entering the room to collect consumables or equipment for their consultations.

Equipping all consulting rooms with computers and user-friendly interfaces for accessing patient information for all clinicians including CNPs is also recommended. This would likely reduce the number of interruptions to request MOs to assist with online enquiries for other patients. Protected administrative time for MOs would also decrease interruptions because of work related to other patients. The design and implementation of electronic health records as outlined in the national eHealth strategy could also improve the efficiencies of the patient-related administrative work in the consultation room, as all patient information (clinical notes, laboratory results, x-rays, discharge information) would be centralised and accessible in one electronic interface.^33,34^

The detailed reasons of the interruptions because of paperwork for other patients and interruptions because of non-personal, non-clinical interactions would be helpful to elucidate. A more detailed qualitative study of these types of interruptions would therefore assist with guiding more focused interventions to decrease their prevalence. Finally, an exploration of the patients’ experiences of these interruptions in these settings is recommended.

## Conclusion

Medical officers in the Western Cape experience a high number of interruptions during their consultations. There is a wide range of interruptions including interruptions not related to the patient in the consultation room. Most notable are avoidable interruptions to collect consumables and interruptions to perform administrative tasks for other patients. Given the short length of consultations in these settings, these interruptions likely affect the quality of care provided to the patients in the consulting rooms through the cognitive disruptions they cause for the doctors. These disruptions can lead to errors which compromise quality of care and patient safety. Organisational interventions like improved equipping of consultation rooms with consumables and electronic equipment like computers and network points are needed, with budget implications. Electronic records with user-friendly input interfaces could also decrease the duration of interruptions to attend to completing the patient’s record during a consultation.
